# Feedback mechanisms control coexistence in a stem cell model of acute myeloid leukaemia

**DOI:** 10.1016/j.jtbi.2016.04.002

**Published:** 2016-07-21

**Authors:** Helena L. Crowell, Adam L. MacLean, Michael P.H. Stumpf

**Affiliations:** Theoretical Systems Biology, Department of Life Sciences, Imperial College London, London SW7 2AZ, UK

**Keywords:** Dynamical systems, Stability analysis, Haematopoietic stem cells, Cancer, Acute myeloid leukaemia

## Abstract

Haematopoietic stem cell dynamics regulate healthy blood cell production and are disrupted during leukaemia. Competition models of cellular species help to elucidate stem cell dynamics in the bone marrow microenvironment (or niche), and to determine how these dynamics impact leukaemia progression. Here we develop two models that target acute myeloid leukaemia with particular focus on the mechanisms that control proliferation via feedback signalling. It is within regions of parameter space permissive of coexistence that the effects of competition are most subtle and the clinical outcome least certain. Steady state and linear stability analyses identify parameter regions that allow for coexistence to occur, and allow us to characterise behaviour near critical points. Where analytical expressions are no longer informative, we proceed statistically and sample parameter space over a coexistence region. We find that the rates of proliferation and differentiation of healthy progenitors exert key control over coexistence. We also show that inclusion of a regulatory feedback onto progenitor cells promotes healthy haematopoiesis at the expense of leukaemia, and that – somewhat paradoxically – within the coexistence region feedback increases the sensitivity of the system to dominance by one lineage over another.

## Highlights

• Models of competition between cell populations can describe the progression of acute myeloid leukaemia.
• We identify regions of coexistence in which leukaemia and healthy haematopoietic species can coexist in the niche.
• The dynamics of progenitor cells exert key control over species coexistence.
• The introduction of regulatory feedback can promote healthy haematopoiesis and suppress leukaemia.

## 1 Introduction

Acute myeloid leukaemia (AML) is a cancer of the blood that causes expanded clones in the myeloid lineage, disrupting healthy haematopoiesis (Löwenberg et al., 1999). Healthy haematopoiesis is governed by a population of haematopoietic stem cells (HSCs), which reside in a stem cell niche within the bone marrow (Wang and Wagers, 2011), and are responsible for the production of all red blood cells, white blood cells, and platelets (Orkin and Zon, 2008). HSCs constitute a rare population of haematopoietic cells, and, through successive symmetrical or asymmetric divisions, they can lose their capacity for unlimited self-renewal and become lineage-restricted committed progenitor cells, before they eventually become terminally differentiated and specialised. This hierarchical organisation helps to protect against malignant transformation within haematopoietic cell lineages.

The cancer stem cell theory proposes that only a subpopulation of cancer cells are responsible for cancer growth and have the capacity to metastasise; they may also be resistant to treatment. This population is referred to as cancer stem cells, and shares characteristics with its healthy counterpart stem cell population in various tissues (Dean et al., 2005). The cancer stem cell theory has, however, been contentious at times, as functional and molecular characterisation of cancer stem cells remains elusive (Woll et al., 2014, Clevers, 2011).

Only a subpopulation of leukaemia cells have the ability to reconstitute the disease following transplantation; we assume in this work that these are cancer stem cells and refer to them as leukaemia stem cells (LSCs). Their existence was first demonstrated by Lapidot et al. (1994) in AML. Later studies found further evidence for the hierarchical organisation of AML (Bonnet and Dick, 1997, Cozzio et al., 2003). Important questions regarding LSCs include whether they are indeed rare (Lane et al., 2009, Quintana et al., 2008), and whether they most closely resemble HSCs or a haematopoietic progenitor cell population (Passegué et al., 2003, Goardon et al., 2011, Lane et al., 2011, Cabezas Wallscheid et al., 2013). It has been shown mathematically that LSCs could comprise any fraction of a blood cancer (Johnston et al., 2010).

A further question, which we believe is fundamental to understanding cancer progression, regards how cancer interacts and competes with the healthy populations in its surroundings. Here, limited experimental research exists (Schuurhuis et al., 2013), and mathematical modelling helps us to address mechanisms of disease and make predictions. Several models have been developed to study leukaemia in general (Roeder and Loeffler, 2002, Michor et al., 2005, Roeder et al., 2006, Horn et al., 2013, Foo et al., 2009, Tang et al., 2011, MacLean et al., 2014, Colijn and Mackey, 2005, Moore and Li, 2004, Werner et al., 2013), and AML in particular (Andersen and Mackey, 2001, Liso et al., 2008, Cucuianu and Precup, 2010, Stiehl and Marciniak-Czochra, 2012, Stiehl et al., 2014). Stiehl et al. (2014) present an attractive model of acute leukaemias, and demonstrate the importance of parameters controlling self-renewal in both diagnosis and relapse. Here, we use a similar modelling framework however our goals and subsequent methods of analysis are different. We seek to characterise how competition processes between healthy and leukaemia stem cells affect species coexistence and disease outcome. The cancer stem cell hypothesis forms a key assumption of our work here, as does the hypothesis that an ecological niche description is required to understand cell interactions within the bone marrow microenvironment.

Certain studies have suggested that the LSC population within AML often shares more features with a progenitor cell population than a stem cell population (Passegué et al., 2003, Goardon et al., 2011), although it is also possible that both stem-like and progenitor-like leukaemia populations coexist (Cabezas Wallscheid et al., 2013). Until recently, little was known about the population dynamics of specific haematopoietic lineages during the progression of AML: this is changing. Cabezas Wallscheid et al. (2013) show that, following expression of the oncogenic fusion protein AML1-ETO, haematopoietic cell lineages are disrupted in particular ways during the path towards leukaemia. A loss of lymphocytes and erythrocytes is accompanied by a dramatic rise in the size of myeloid populations. In the more primitive haematopoietic compartments, changes to population size were not seen: the leukaemic transformation events take place in primitive stem and progenitor cell compartments, affecting the myeloid and lymphoid progeny.

We seek to understand in greater depth the shape of competition during disease progression by modelling the interactions and feedbacks between leukaemia and haematopoietic species, specifically, we model competition occurring between LSCs and healthy progenitor cells. In addition to the role that progenitors play in leukaemia, there is growing evidence that this population plays a greater role in haematopoiesis than had previously been assumed, promoting the idea that a renewed focus on the dynamics of haematopoietic progenitor cells is warranted (Sun et al., 2014). Recent work has shown how AML disrupts haematopoiesis by forming malignant niches capable of sustaining disease, highlighting leukaemia's ability to dramatically affect haematopoietic niches (Hanoun et al., 2014). Based on previous work that provided insight into competition within the HSC niche (MacLean et al., 2013), here we develop two new models that differ in their treatment of signalling between progenitor and terminally differentiated haematopoietic cells.

In the next section we introduce the models and describe their basic properties. We go on to analyse model behaviour using a combination of analytical and numerical techniques, to identify what factors control the competition between LSCs and progenitor cells. We are particularly interested in those regions of behaviour space that allow for coexistence between leukaemia and haematopoietic species, as these are most crucial in determining clinical outcome.

## 2 Competition models of acute myeloid leukaemia

Two new models are proposed that each describes the dynamics of AML in the bone marrow. They differ subtly, regarding mechanisms of feedback that we wish to compare. Each contains five cellular species, the dynamics of which are described by ordinary differential equations (ODEs).

Competition models are based upon the ideas introduced by Lotka and Volterra and later ecologists (Lotka, 1920, Volterra, 1926, May and MacArthur, 1972). Ecological concepts can also be applied in a cellular context, such as within the stem cell niche (MacLean et al., 2013, Mangel and Bonsall, 2013). Here, competing species share a reliance on finite environmental resources including nutrients, cofactors, and molecular signals which are essential for their functionality. Even though we expect feedback effects to increase faster as competing species accumulate — i.e. crowdedness within the niche amplifies regulatory signals, — we assume them to be (i) *linear* and (ii) *proportional* to the population sizes of all species involved.

The diverse types of blood cells encountered in the body are derived from a self-renewing population of haematopoietic stem cells (HSCs – or species *S* in the model), which can differentiate into multipotent progenitor cells, and eventually terminally differentiated cells. Given that we focus on the dynamics of differentiation and blood cell production, we group the various haematopoietic species into two populations: haematopoietic progenitor cells (*A*), and specialised, terminally differentiated blood cells (*D*), similar to previous work (MacLean et al., 2013).

In the following models AML consists of two distinct cell populations: a proliferating leukaemia cell population (*L*); and a population of terminally differentiated leukaemia cells (*T*). Proliferating leukaemia cells are assumed to be in competition with haematopoietic progenitor cells, rather than HSCs. Thus HSC dynamics are not directly impacted by AML, although there will be an indirect effect through feedback. Although we refer to population *L* as leukaemia stem cells (LSCs), this does not refer to their cell of origin, but only to their lineage-maintaining characteristics (Dick, 2008). Additionally, in this work we consider questions about cancer progression, and leave the matter of cancer incidence for elsewhere.

### 2.1 Model I

**Fig. 1:**
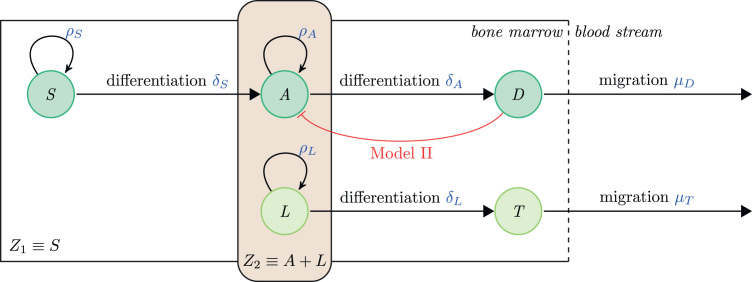
Mechanistic model of the interacting species. Upper row: haematopoietic linage. Haematopoietic stem cell (*S*), progenitor blood cell (*A*) and differentiated blood cell (*D*). Lower row: leukaemia linage. Leukaemia stem cell (*L*) and mature leukaemia cell (*T*). Healthy progenitors and LSCs occupy and compete for the same ecological niche (rounded box) within the bone marrow. Differentiated blood- and mature leukaemia cells migrate into the bloodstream. Negative feedback control indicated in red. (For interpretation of the references to colour in this figure caption, the reader is referred to the web version of this paper.)

We describe the dynamics of the five species introduced above with a system of ODEs. A schematic description of the Model I is given in Fig. 1; and the model is specified by the following equations:

(1a) $$\frac{\mathrm{dS}}{\mathrm{dt}}=\rho_{S}S(K_{1}-Z_{1})-\delta_{S}S$$

(1b) $$\frac{\mathrm{dA}}{\mathrm{dt}}=\delta_{S}S+\rho_{A}A(K_{2}-Z_{2})-\delta_{A}A$$

(1c) $$\frac{\mathrm{dD}}{\mathrm{dt}}=\delta_{A}A-\mu_{D}D$$

(1d) $$\frac{\mathrm{dL}}{\mathrm{dt}}=\rho_{L}L(K_{2}-Z_{2})-\delta_{L}L$$

(1e) $$\frac{\mathrm{dT}}{\mathrm{dt}}=\delta_{L}L-\mu_{T}T$$

where total niche sizes $Z_{1}=S$ and $Z_{2}=A+L$, and species phenotypes are specified by the rates *ρ*_*i*_ (proliferation), *δ*_*i*_ (differentiation), and *μ*_*j*_ (migration), with i∈{S,A,L} and j∈{D,T}. Definitions of the model parameters are given in Table 1. The population sizes of cells within the bone marrow are set by the carrying capacities, *K*_1_ and *K*_2_. For simulations of the model, we scale the populations sizes such that $K_{1}=K_{2}=1$.

### 2.2 Model II

The species engaged in competition and the definition of niches remain unaltered in Model II, however it incorporates a new feedback response from terminally differentiated blood cells (*D*) onto the progenitor cell population *A*, such that as the population size of the differentiated blood cells increases, the transition rate *δ*_*A*_ (A→D) decreases.

Thus, Eqs. (1a), (1d), (1e) are equal to their equivalents for Eqs. (2a), (2b), (2c), (2d), (2e), and the full Model II is specified by the following equations:

(2a) $$\frac{\mathrm{dS}}{\mathrm{dt}}=\rho_{S}S(K_{1}-Z_{1})-\delta_{S}S$$

(2b) $$\frac{\mathrm{dA}}{\mathrm{dt}}=\delta_{S}S+\rho_{A}A(K_{2}-Z_{2})-\frac{\delta_{A}A}{1+D}$$

(2c) $$\frac{\mathrm{dD}}{\mathrm{dt}}=\frac{\delta_{A}A}{1+D}-\mu_{D}D$$

(2d) $$\frac{\mathrm{dL}}{\mathrm{dt}}=\rho_{L}L(K_{2}-Z_{2})-\delta_{L}L$$

(2e) $$\frac{\mathrm{dT}}{\mathrm{dt}}=\delta_{L}L-\mu_{T}T$$

where the parameters are defined as for Model I (Table 1) and the additional interactions of this model can be seen in Fig. 1.

In summary, both models consider how competition between *A* and *L* affects lineage maintenance, in the absence (Model I) and presence (Model II) of lineage-mediated regulatory feedback. As seen from the definition of *Z*_2_, only species *A* and *L* are niche effectors, however their progeny can have indirect effects. Introduction of negative feedback within the haematopoietic hierarchy in Model II is — as expected and will be shown later — advantageous, and increases the propensity of healthy progenitors outcompeting their competitors.

These models incorporate our current understanding of the potential interactions between the healthy and malignant haematopoietic systems. And their simplicity eschews making extraneous, poorly supported assumptions that could bias our analyses. The models serve as mean-field approximations of niche dynamics, where we would expect spatial dependencies to arise from the cell–cell interactions that are believed to shape the behaviour in the niche.

## 3 Results

We begin with an analysis of the solutions to Eqs. (1a), (1b), (1c), (1d), (1e) for Model I and Eqs. (2a), (2b), (2c), (2d), (2e) for Model II, starting with the stationary states. When analytical analysis becomes intractable, we appeal to statistical approaches for further investigation of competition within the stem cell niche.

### 3.1 Steady state analysis for Model I

The steady states of Model I are specified by,

(3a) $$S^{⋆}=1-\frac{\delta_{S}}{\rho_{S}}$$

(3b) $$A^{⋆}=-\frac{\delta_{S}S^{⋆}}{\frac{\rho_{A}\delta_{L}}{\rho_{L}}-\delta_{A}}$$

(3c) $$D^{⋆}=\frac{\delta_{A}A^{⋆}}{\mu_{D}}$$

(3d) $$L^{⋆}=1-A^{⋆}-\frac{\delta_{L}}{\rho_{L}}$$

(3e) $$T^{⋆}=\frac{\delta_{L}L^{⋆}}{\mu_{T}}.$$

**Fig. 2:**
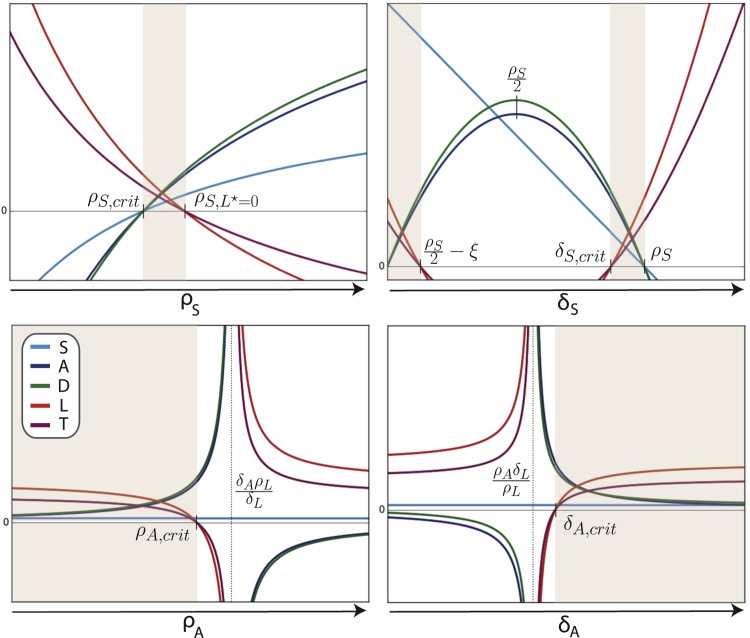
Steady states of Model I for species *S*, *A*, *D*, *L* and *T* over parameters *ρ*_*S*_, *δ*_*S*_, *ρ*_*A*_ and *δ*_*A*_, respectively. Remaining parameters are held at fixed values. Asymptotic and critical values are indicated; shaded regions denote coexistence.

Steady state species population sizes for varying rates are shown in Fig. 2. These provide us with a better understanding of the influence of each parameter on the system and help to elucidate characteristic values that define qualitatively distinct regions in parameter space. We focus particularly on regions where $A^{⋆}>0$ and $L^{⋆}>0$. An overview of steady state characteristics is given in Table 2.

Upon inspection of the reliance of the steady states on *δ*_*S*_ (HSC differentiation), we see that *A* and *D* assume maximal steady state populations when $\delta_{S}=\frac{\rho_{S}}{2}$. At this point $L^{⋆}$ and $T^{⋆}$ are minimised. These optimal population sizes for *A* and *D* thus arise when HSC proliferation occurs at double the rate of HSC differentiation, independent of all other parameters.

Regions permissive of coexistence of healthy and leukaemia lineages are indicated by the shaded regions in Fig. 2, and are defined by the boundary conditions,

$$\rho_{S,\mathrm{crit}}=\delta_{S}\rho_{S,L^{⋆}=0}=\frac{\delta_{S}}{1-\frac{\left(\frac{\delta_{L}}{\rho_{L}}-1)(\frac{\rho_{A}\delta_{L}}{\rho_{L}}-\delta_{A}\right)}{\delta_{S}}}$$

and

$$\delta_{S,\mathrm{crit}}=\frac{\rho_{2}}{2}+\xi \delta_{S}=\rho_{S}$$

where

$$\xi^{2}=\frac{\rho_{S}^{2}}{4}+\rho_{S}(\frac{\delta_{L}}{\rho_{L}}-1)(\frac{\rho_{A}\delta_{L}}{\rho_{L}}-\delta_{A}),$$

$\rho_{S,\mathrm{crit}}$ denotes the value for *ρ*_*S*_ such that $S^{*}=0$ (similarly for $\delta_{S,\mathrm{crit}}$), and $\rho_{S,L^{*}=0}$ denotes the value of *ρ*_*S*_ such that $L^{*}=0$. As a consequence, the range of parameters allowing for species to coexist is also confined. Crossing these boundaries will favour one species over the other to a point where the subordinate lineage will go extinct.

In contrast, proliferation and differentiation rates for the progenitor cells appear to be less restrictive; coexistence is possible for all parameter combinations below the critical value for *ρ*_*A*_ and above the critical value for *δ*_*A*_, where

$$\rho_{A,\mathrm{crit}}=\frac{\rho_{L}}{\delta_{L}}(\frac{\delta_{S}S}{\frac{\rho_{L}}{\delta_{L}}-1}+\delta_{A})\delta_{A,\mathrm{crit}}=\frac{\rho_{A}\delta_{L}}{\rho_{L}}-\frac{\delta_{S}S}{\frac{\delta_{L}}{\rho_{L}}-1}.$$

Robustness of the stem cell pool to changes in progenitor cell dynamics can be seen in the bottom two panels of Fig. 2, where *S* is fixed at a constant value. The healthy progenitor lineage has a greater capacity for (re)generation, given contributions from both self-renewal and production from stem cells; this might explain the larger regions of coexistence that are seen for changes in progenitor dynamics in comparison with changes in stem cell dynamics.

### 3.2 Steady state analysis for Model II

The steady states for Model II are given by Eqs. (3a), (3d), (3e) and the following,

(4a) $$A^{⋆}=\frac{\mu_{D}D^{*}(1+D^{*})}{\delta_{A}}$$

(4b) $$D^{⋆}=\frac{\frac{\delta_{A}\rho_{L}}{\rho_{A}\delta_{L}}-1}{2}\pm \sqrt{\frac{{\left(\frac{\delta_{A}\rho_{L}}{\rho_{A}\delta_{L}}-1\right)}^{2}}{4}-\frac{\delta_{S}\delta_{A}\rho_{L}S^{*}}{\rho_{A}\delta_{L}\mu_{D}}}$$

that is, changing the form of the feedback affects only the steady state solutions for healthy progenitor and differentiated blood cells.

**Fig. 3:**
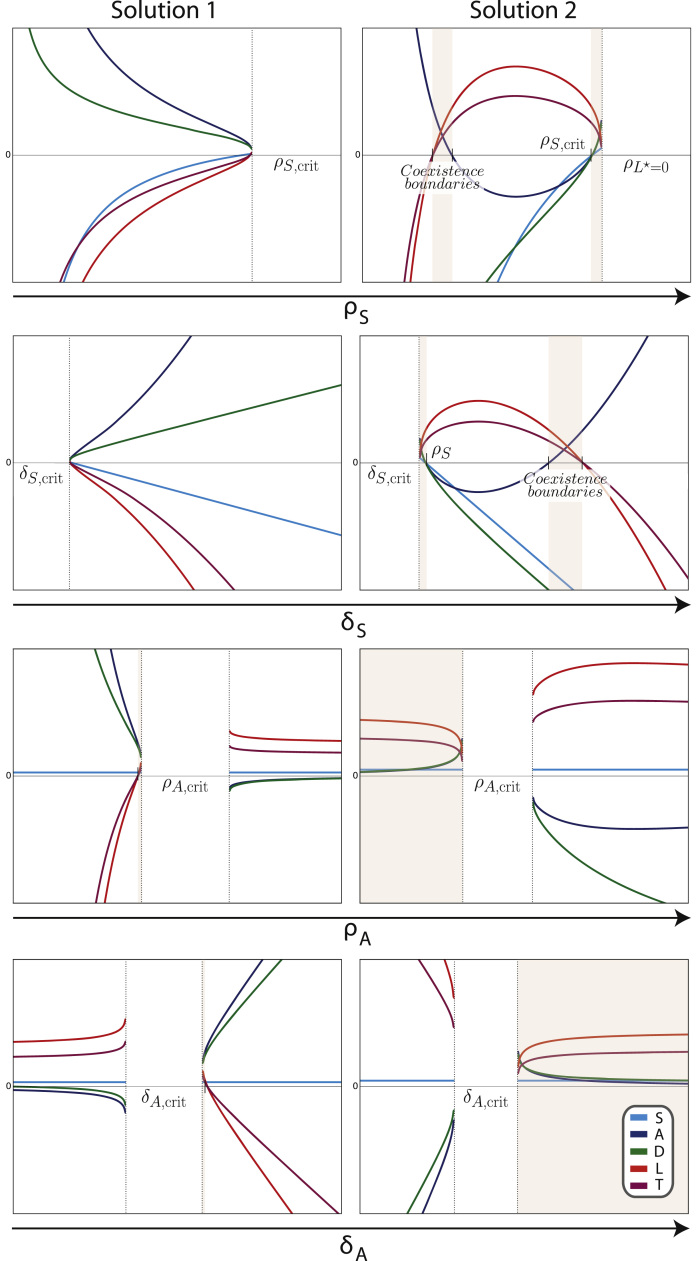
Steady states of Model II for species *S*, *A*, *D*, *L* and *T* over parameters *ρ*_*S*_, *δ*_*S*_, *ρ*_*A*_ and *δ*_*A*_, respectively. Two solutions exist for each species. Asymptotic or critical values are indicated when solutions are real numbers. Dotted lines denote boundaries where at least one species' steady state becomes complex: no species are plotted in the regions where their solutions are complex; and critical values that lie within these regions are marked but left unspecified. Shaded regions denote coexistence.

The species' population sizes at steady state are shown in Fig. 3. Characterising the steady state behaviour of Model II analytically proves to be difficult. Solutions yield imaginary results, so the calculation of biologically motivated critical values becomes impossible.

We still can, however, gain some information from studying the analytical steady states of Model II. In Fig. 3 we see that for the first solution (left hand column) there are no coexistence regions with real values, but for the second solution (right hand column) there are coexistence regions: the parameter most permissive of coexistence over this range is *δ*_*A*_, whereas for *ρ*_*S*_ and *δ*_*S*_ we have much more narrow regions permitting coexistence.

### 3.3 Linear stability analysis in regions of coexistence

In order to further characterise the behaviour of these models, we can study the asymptotic stability of the fixed points (steady states) of the system, and investigate whether a model is (locally) stable to small perturbations around that fixed point (Strogatz, 1994). As we have seen in the previous sections, steady states exist for which only healthy species or leukaemia species have positive population sizes, and steady states also exist where both healthy and leukaemia species can coexist. Since we are most interested in the behaviour of these coexistence regions, we focus our stability analyses on these regions.

A fixed point is stable for a given set of parameters values if all the eigenvalues of the matrix $\partial f_{i}/\partial x_{j}$ are negative (Strogatz, 1994), where $x_{j}\in (S,A,D,L,T)$ and the *f*_*i*_ correspond to the right hand side of Eqs. (1a), (1b), (1c), (1d), (1e) for Model I, or (2a), (2b), (2c), (2d), (2e) for Model II. We begin by choosing a point in the coexistence region to study, where $\left(\rho_{S},\rho_{A},\delta_{S},\delta_{A})=(0.5,0.43,0.14,0.44\right)$; see Section 3.4 for a discussion of the parameter values used. Under these conditions, the fixed point for Model I, and one of the fixed points for Model II show coexistence of healthy and leukaemia species, and the state is locally asymptotically stable.

Given the uncertainty in parameter values, it is much more informative to study the stability properties of a steady state of a model within some parameter range, rather than for single values (Kirk et al., 2015). We have thus analysed for 10,000 parameters sets (each parameter sampled in the range [0.1,0.5]) the fraction of the resulting fixed points that are stable, within the coexistence regions for each model (see Fig. 2, Fig. 3). For the fixed point of Model I, the fraction of stable states within the coexistence region is 0.359. For the first fixed point of Model II, the corresponding fraction of stable states is 0.115. For the second fixed point of Model II, no solutions within the coexistence region are stable. Steady states of Model I within the coexistence region are thus more likely to be stable than equivalent states within the coexistence region of Model II.

Further analytical characterisation of the models is impractical, and in order to proceed with analysis of the models in more depth, and to draw comparisons between the models, we appeal to statistical methods that allow us to numerically simulate and analyse model behaviour over regions of parameter space.

### 3.4 Exploration of model characteristics in parameter space

Preliminary experimental studies have begun to elucidate how AML progresses within the HSC niche and disrupts haematopoiesis (Hu et al., 2009, Lane et al., 2011, Miraki-Moud et al., 2013, Cabezas Wallscheid et al., 2013). While these studies are tantalising (for example, estimation of population growth rates from the data provided in Cabezas Wallscheid et al. (2013) may be possible), they describe only the coarse-grained dynamics of healthy and malignant haematopoietic species. In Stiehl et al. (2015), further progress towards characterising the dynamics of AML progression is made; these results also highlight the level of variability in cell population responses. Frank appraisal of this variability, and accounting for the uncertainty present in such data, requires methods that explore regions of parameter space rather than use single parameter values (MacLean et al., 2013, Gutenkunst et al., 2007, Komorowski et al., 2011).

Here we focus on the parameters of healthy haematopoiesis and hold fixed those parameters controlling leukaemia species. We do so in order to investigate relative rates of haematopoiesis, given constant rates of leukaemia species production. We set the clearance rates of differentiated healthy (*D*) and leukaemia (*T*) cells to $\mu_{D}=0.275$ and $\mu_{T}=0.3$, such that the leukaemia cells are slightly shorter-lived in the system. We counterbalance this by a proliferation rate of LSCs that is higher than their differentiation rate: $\rho_{L}=0.27$ and $\delta_{L}=0.2$. We are then left with a four-dimensional parameter space consisting of ($\rho_{S},\delta_{S},\rho_{A},\delta_{A}$). We vary each of these parameters within the range [0.1, 0.5]. This enables analysis of behaviour in a region of parameter space that corresponds to rich dynamical behaviour.

In total, we consider approximately three million parameter combinations, and we study three qualitative features with which we can characterise behaviour, namely: (i) $A^{⋆}>0$ or $L^{⋆}>0$, i.e. progenitor cells or LSCs reach positive steady states; (ii) coexistence of species *A* and *L*; (iii) given coexistence of *A* and *L*, *A* dominates over *L*, or vice versa. We simulate each ODE system using an implementation of the LSODA numerical solver (in Python 2.7), which is well-suited for stiff ODE systems.

**Fig. 4:**
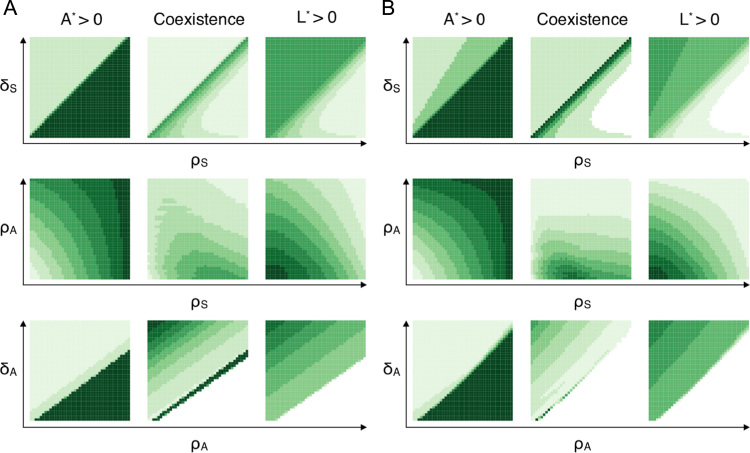
Density plots correlating parameters *ρ*_*S*_, *ρ*_*A*_, *δ*_*S*_ and *δ*_*A*_. Model I (A) and Model II (B). Each pair of values is represented by a two-dimensional bin with brightness corresponding to count: form zero (white) to a maximum (dark green): the darkest regions highlight where features are most frequently observed. (For interpretation of the references to colour in this figure caption, the reader is referred to the web version of this paper.)

Fig. 4 comprises 2D density plots of features (i) and (ii), where the density is calculated from binning the proportion of parameter pairs displaying a certain feature over all parameter pairs. We study the effects that parameter variation has upon the frequency of these features for Model I (Fig. 4A) and Model II (Fig. 4B). Parameter correlations can be classified into two groups,

1. Parameter pairs ($\rho_{S},\delta_{S}$) and ($\rho_{A},\delta_{A}$) demonstrate sharp changes in density. At certain ratios for these pairs, feature counts drop to zero (e.g. crossing the line $\rho_{A}>\delta_{A,c}$ for increasing *ρ*_*A*_), or increase significantly.
2. For the remaining set of parameter pairs: ($\rho_{S},\rho_{A}$), ($\rho_{S},\delta_{A}$), ($\delta_{S},\rho_{A}$) and ($\delta_{S},\delta_{A}$), such sharp changes in density are not seen. For these pairs, density changes occur in a more gradual fashion, where density varies continuously from low to high.

There exist regions in Fig. 4 of constant density; within these the system is robust to changes in the features of interest. For example, for all parameter sets satisfying $\frac{\rho_{S}}{\delta_{S}}>1$, progenitor cells have a high and constant probability of taking positive steady state values that is independent of their precise proliferation rate.

For Model I, progenitor cells appear to be very likely to win over LSCs as long as $\rho_{S}>\rho_{S,\mathrm{crit}}$, whereas they are likely to go extinct for $\rho_{S}<\rho_{S,\mathrm{crit}}$ (Fig. 4A, top row). So in order to maintain a pool of progenitors cells, it is almost sufficient for the rate of stem cell self-renewal to exceed their rate of differentiation into progenitors. Similar conclusions can be drawn for ($\rho_{A},\delta_{A}$): sets for which $\rho_{A}>\rho_{A,c}$ (or, equivalently, $\delta_{A}<\delta_{A,c}$) are likely to yield positive steady states for *A*; whereas reaching positive steady states for *A* is unlikely for smaller *ρ*_*A*_ (larger *δ*_*A*_) (Fig. 4A, bottom row). Above $\rho_{A,c}$ or below $\delta_{A,c}$, the frequency of $A^{⋆}>0$ becomes independent of these rates. In other words, a sufficiently high rate of self-renewal of progenitor cells (or a sufficiently small differentiation rate) almost surely gives rise to a population of progenitor cells at steady state. Parameters controlling values that are likely to give rise to the existence of *L* satisfy converse conditions. Of note, the ratio $\frac{\rho_{A}}{\delta_{A}}$ allows for a positive progenitor population ($A^{*}>0$) over a larger range of values for Model I than it does for Model II (Fig. 4A and B, bottom rows).

The probability of species' coexistence can be represented as the frequency of observing $A^{⋆}>0$ and $L^{⋆}>0$. As specified in Table 2, steady state solutions for *A* and *L* detail boundary conditions that define a constrained zone within parameter space where species may coexist. In particular, we observe a narrow region of the highest coexistence probability around $\rho_{S}=\delta_{S}$. Once a critical $\frac{\rho_{A}}{\delta_{A}}$ value is exceeded, i.e. $\rho_{A}>\rho_{A,\mathrm{crit}}$ or $\delta_{A}<\delta_{A,\mathrm{crit}}$, leukaemia stem cells are driven to zero and species coexistence becomes impossible. Both species may coexist for smaller *ρ*_*A*_; here there is a high density region corresponding to certain survival of *L* that decreases as $\frac{\rho_{A}}{\delta_{A}}$ approaches its critical value.

**Fig. 5:**
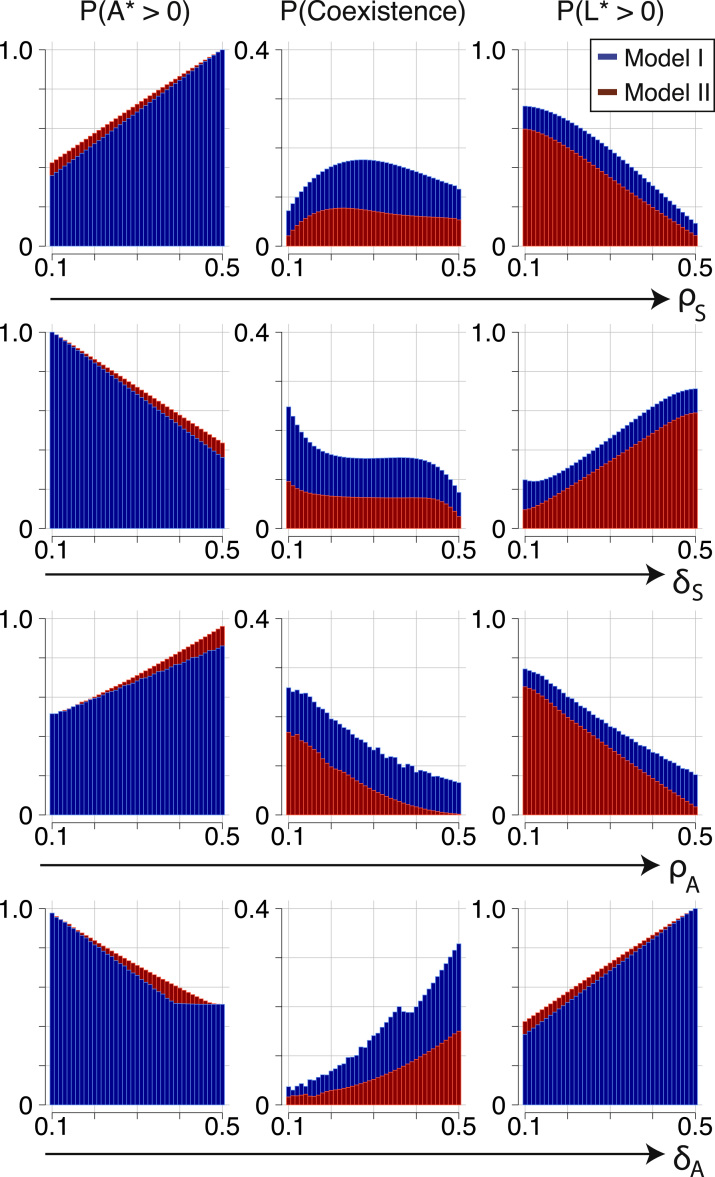
Histograms summarise the probability of observing three features of interest over a range of parameter values for *ρ*_*S*_, *ρ*_*A*_, *δ*_*S*_ and *δ*_*A*_. $A^{*}(L^{*})$ denotes the value of species A(L) at steady state.

The presence or lack of coexistence appears to be dictated predominately by the dynamics of the haematopoietic lineage, specifically, whether *ρ*_*S*_ and *δ*_*S*_ fall within an interval that is permissive of coexistence (see the parameter sensitivity observed in Fig. 4, top rows). However, within such an interval, the rates controlling progeny species become important and have a significant influence upon the probability of species coexistence. This is summarised in Fig. 5. The probability of coexistence increases with increasing *δ*_*A*_, and decreases with increasing *ρ*_*A*_. In line with this, while moving towards a maximal $\frac{\delta_{A}}{\rho_{A}}$ ratio in Fig. 4 (bottom row), the density increases. In comparison, the joint distribution over ($\delta_{S},\rho_{S}$) features a single high density region around $\rho_{S}=\delta_{S}$ and at points on the joint space further away from here the probability of coexistence is relatively insensitive to changes in the parameters.

From Fig. 4, we see that to a certain extent the coexistence features of Model I are shared by Model II. They differ where, for example, under Model II, *L* is certain (rather than likely) to go extinct (Fig. 4B, top row). This is in accordance with a more restricted parameter space allowing coexistence for Model II. Furthermore, a greater area of the joint density space of ($\rho_{S},\delta_{S}$) permits $A^{⋆}>0$. Progenitor blood cells are consequently found to have a higher probability of survival whereas survival of leukaemia is in general less likely, and indeed (as already mentioned) impossible for a large range of parameters.

We can consider the gradient of the density distributions as a measure of the robustness or sensitivity of the system to particular changes in parameters, with steeper gradients corresponding to more sensitive regions. For example, for Model I, the probability that $A^{*}>0$ increases as *ρ*_*S*_ and *ρ*_*A*_ increase, and as *δ*_*S*_ and *δ*_*A*_ decrease; the reverse trend can be seen for $L^{*}>0$ (Fig. 4A and B, middle and bottom rows). Thus both of these features are sensitive to such changes in the parameters. The progenitor cell rates *ρ*_*A*_ and *δ*_*A*_ also affect the probability that species will coexist. For Model II, coexistence appears to be robust to varying *ρ*_*S*_ and *δ*_*S*_; in contrast for Model I, the probability of coexistence follows a parabolic curve, culminating at $\rho_{S}=\rho_{S,\mathrm{crit}}=\delta_{S}$.

Furthermore, as parameter *δ*_*A*_ varies, the probabilities that $A^{⋆}>0$, coexistence occurs, and $L^{⋆}>0$ all feature non-smooth transitions in the region $\left[\delta_{A_{1}},\delta_{A_{2}}\right]$. These can be defined as

$$\delta_{A_{1}}=\delta_{A,\mathrm{asymp}}\;\mathrm{such}\;\mathrm{that}\;\rho_{A}=0.5,$$

and

$$\delta_{A_{2}}=\delta_{A,\mathrm{crit}}\;\mathrm{such}\;\mathrm{that}\;\rho_{A}=\delta_{S}=0.1\;\mathrm{and}\;\rho_{S}=0.5.$$

where $\delta_{A,\mathrm{crit}}$ is (as previously), the value of *δ*_*A*_ for which $A^{*}=0$, and $\delta_{A,\mathrm{asymp}}$ is the asymptotic value for which $A^{*}\to \infty$. Since $A^{*}=0$ for $\delta_{A}\geq \delta_{A,\mathrm{crit}}$, $P(A^{⋆}>0)$ is unaffected by changes in *δ*_*A*_ for $\delta_{A}\geq \delta_{A_{2}}$. In addition we see that the coexistence probability is dependent on both $\delta_{A_{1}}$ and $\delta_{A_{2}}$, and is independent of *δ*_*A*_ for $\delta_{A_{1}}<\delta_{A}<\delta_{A_{2}}$.

The presence of negative feedback within the haematopoietic linage clearly favours the odds of survival of healthy progenitors. Leukaemia stem cells are more likely to go to zero at steady state, for any given parameter values. These results are quantified in Table 3; notably, the largest difference between models is the probability of continuing existence of leukaemia stem cells.

**Fig. 6:**
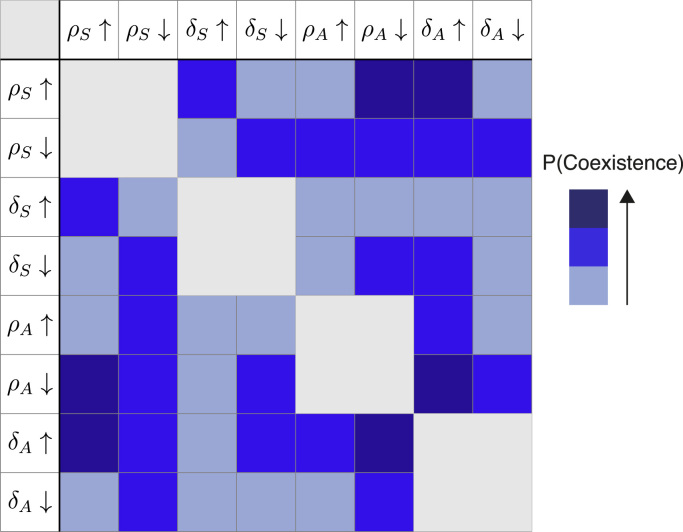
Dominance of species within the coexistence region of parameter space.

Fig. 6 presents an overview of the results of this section. It does not contain the finer details notable in Fig. 4, Fig. 5, but provides an overview of the more salient features observed, and can be used to see how high or low-valued parameter pairs affect the probability of coexistence.

### 3.5 Further characterisation of coexistence regions

**Fig. 7:**
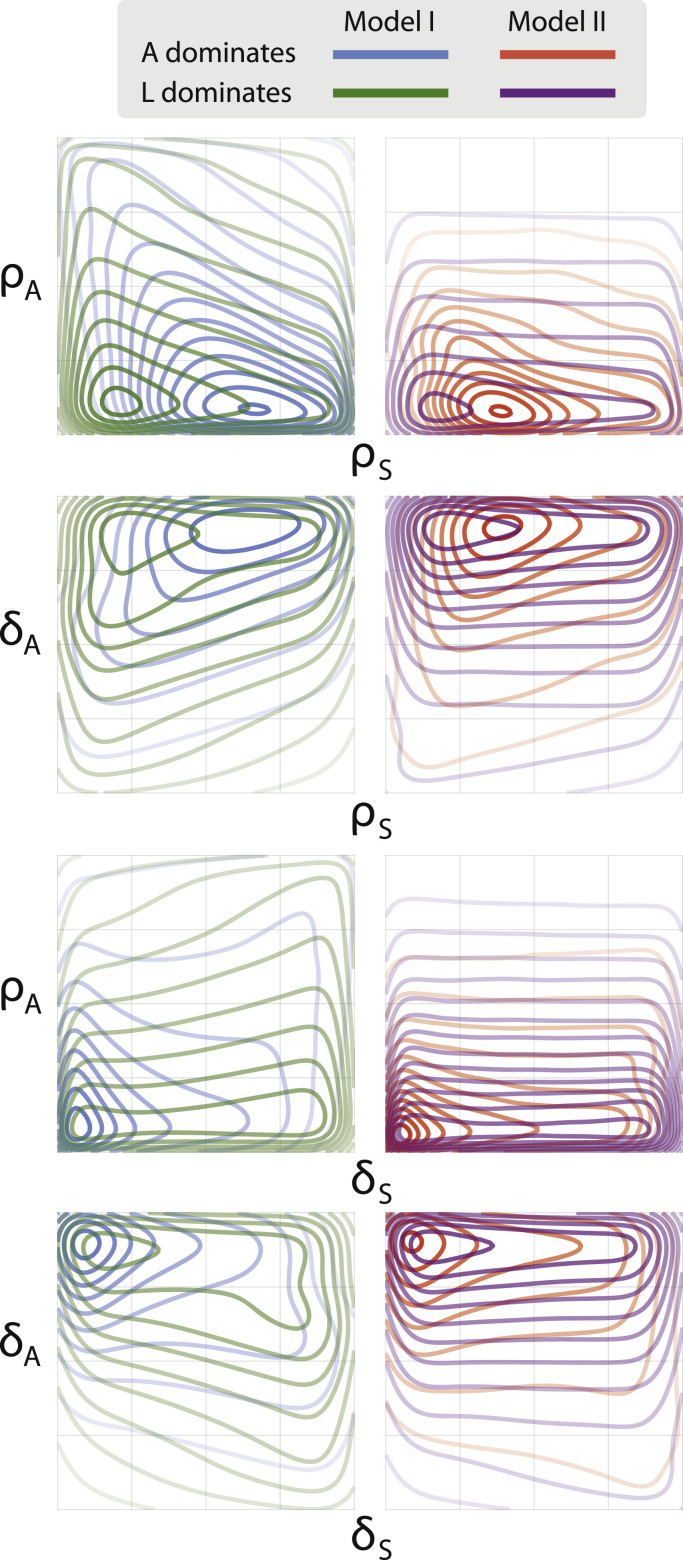
Dominance of species within the coexistence region of parameter space.

In our exploration of the models' behaviour, we have identified coexistence regions and found that they occur only in restricted areas of parameter space. Such regions are of particular interest because they allow us to make predictions about cancer progression when the outcome is least certain. We argue that such situations, when there is no obvious dominance of cancer species over their native counterparts, and the behaviours of the different lineages are (at least transiently) similar, might occur more frequently during AML progression or treatment than often considered. In order to probe in greater detail the interplay of species during coexistence, Fig. 7 studies the probability that one or the other lineage will dominate, given coexistence.

The same critical values defined above apply here, so for example, dominance of *L* is highly improbable if $\rho_{S}>\rho_{S,\mathrm{crit}}=\delta_{S}$. Furthermore, we note that fewer parameters are permissive of coexistence for Model II, as can be seen from the resulting density plots for this model. Fig. 7 illustrates that, for both models, coexistence is restricted to small values of *ρ*_*A*_ and large values of *δ*_*A*_. Within this region, for either *ρ*_*A*_ or *δ*_*A*_, similar differences for each model emerge: *ρ*_*S*_ controls which lineage dominates. Higher values of *ρ*_*S*_ support *A* (healthy lineage), whereas lower values of *ρ*_*S*_ support leukaemia. The difference between models here is subtle, but we do see that the separation of disease outcomes is less clear for Model II than Model I. This is intriguing: it indicates that the feedback regulation of Model II — in addition to decreasing the probability of coexistence overall — also increases the sensitivity of the system with respect to lineage domination.

For the rate of HSC differentiation, *δ*_*S*_, we see less variation within models regarding lineage dominance. In Model I, however, there is a larger region where leukaemia is more likely to dominate than the healthy lineage. This restriction of the leukaemia dominance region for Model II corroborates earlier results — that the addition of feedback benefits healthy haematopoiesis, giving HSCs and their progeny a possible advantage over their cancerous counterparts.

## 4 Discussion

In this work we have developed models for AML and used them to map out the effects of competition on cancer progression within the haematopoietic stem cell niche and surrounding environment. AML is an interesting disease in that it is most likely driven by a population of stem-like leukaemia cells which are heterogeneous in nature and might most closely resemble healthy progenitor cells or HSCs (Goardon et al., 2011, Cabezas Wallscheid et al., 2013).

By characterising solutions according to outcomes defining survival and competition, we can compare the likelihood of niche dominance by one lineage over another. Of the two models considered, the first describes simple competition between leukaemia stem cells and healthy progenitor cells, and the second builds on this with the inclusion of feedback that acts to regulate the progenitor cell pool in response to changes in the differentiated cell population size. That is, feedback enables differentiated blood cells to signal to their parent population and transmit information about their population size before leaving the bone marrow and entering the blood stream. This allows haematopoietic progenitor cells to adjust their rate of differentiation to fluctuating demands, and delay their differentiation accordingly. As a result, healthy progenitor cells are bestowed a competitive advantage over leukaemia stem cells.

Significant efforts have been made to understand chronic myeloid leukaemia through modelling, with considerable success (Roeder and Loeffler, 2002, Moore and Li, 2004, Colijn and Mackey, 2005, Michor et al., 2005, Roeder et al., 2006, Foo et al., 2009, Tang et al., 2011, Horn et al., 2013, MacLean et al., 2014). Although the dynamics of acute myeloid leukaemia are similar, they differ in certain key characteristics, including the relative dynamics of myeloid/lymphoid lineages (Cabezas Wallscheid et al., 2013) and the resemblance of leukaemia stem cells to healthy progenitor cells. By incorporating some of these features into a theoretical model, we provide predictions about factors influencing disease outcome in the bone marrow niche during acute leukaemia. Previous work has studied the role of competition in disease progression, and proposed strategies for treatment; here we delve deeper into the mechanisms of competition, particularly within coexistence regions. We obtained analytical results (for Model I), which provide global characterisation of model behaviour at equilibrium, and complement these results with statistical analyses. Intriguingly, the relatively small parameter space in agreement with coexistence (especially for populations experiencing feedback) could be linked to the relatively rarity of AML and its severity upon diagnosis, which could be preceded by a long asymptomatic phase.

A central challenge in stem cell biology is our inability — at least in most cases — to make detailed measurements of processes occurring within physiological niches, especially in sufficient spatiotemporal resolution. (Organoids can offer some insight into aspects of the stem cell dynamics by mimicking tissue in vitro (Sato et al., 2009)). In order to continue to unravel the mechanisms that control stem cell behaviour, and the progression of cancer in blood and solid tissue, hypothesis-generating models accelerate progress by making testable predictions. Through tight coupling of theoretical analysis and experimentation, we can hope to describe more fully the wealth of behaviours that haematopoietic stem cells and their progeny exhibit, and the pathways that regulate incidence and progression of leukaemia.

## Conflict of interest

None declared.

## Figures and Tables

**Table 1:** Parameter set which describes Models I and II.

**Parameter**	**Definition**
*ρ*_*S*_	Proliferation of *S*
*ρ*_*A*_	Proliferation of *A*
*ρ*_*L*_	Proliferation of *L*

*δ*_*S*_	Differentiation of S→A
*δ*_*A*_	Differentiation of A→D
*δ*_*L*_	Differentiation of L→T

*μ*_*D*_	Migration of *D*
*μ*_*T*_	Migration of *T*

**Table 2:** Summary of analytical characteristics in parameter space for Model I.

Parameter	$A^{⋆}>0$	*Coexistence*	$L^{⋆}>0$
*ρ*_*S*_	$>\rho_{S,\mathrm{crit}}$	$\in [\rho_{S,\mathrm{crit}},\rho_{S,L^{⋆}=0}]$	$<\rho_{S,L^{⋆}=0}$
*δ*_*S*_	$<\rho_{S}$	$<\frac{\rho_{S}}{2}-\xi$ or $\in [\delta_{S,\mathrm{crit}},\rho_{S}]$	$\notin [\frac{\rho_{S}}{2}-\xi ,\delta_{S,\mathrm{crit}}]$
*ρ*_*A*_	$<\frac{\delta_{A}\rho_{L}}{\delta_{L}}$	$<\rho_{A,\mathrm{crit}}$	$\notin [\rho_{A,\mathrm{crit}},\frac{\delta_{A}\rho_{L}}{\delta_{L}}]$
*δ*_*A*_	$>\frac{\rho_{A}\delta_{L}}{\rho_{L}}$	$>\delta_{A,\mathrm{crit}}$	$\notin [\frac{\rho_{A}\delta_{L}}{\rho_{L}},\delta_{A,\mathrm{crit}}]$

**Table 3:** Model comparison of features of interest. The probability of observing each feature is given; *C* denotes coexistence.

	($A^{⋆}>0$)	($L^{⋆}>0$)	Coexistence	$(A^{⋆}>L^{⋆})|C$	$(L^{⋆}>A^{⋆})|C$
Model I	0.683	0.466	0.148	0.087	0.061
Model II	0.719	0.344	0.063	0.029	0.034

% Change	+ 3.6	– 12.2	– 8.5	– 5.8	– 2.7
